# Interleukin-10 Mediated Autoregulation of Murine B-1 B-Cells and Its Role in *Borrelia hermsii* Infection

**DOI:** 10.1371/journal.pone.0011445

**Published:** 2010-07-06

**Authors:** Vishal Sindhava, Michael E. Woodman, Brian Stevenson, Subbarao Bondada

**Affiliations:** 1 Department of Microbiology, Immunology and Molecular Genetics, University of Kentucky College of Medicine, Lexington, Kentucky, United States of America; 2 Markey Cancer Center, University of Kentucky College of Medicine, Lexington, Kentucky, United States of America; Agency for Science, Technology and Research (A*STAR), Singapore

## Abstract

B cells are typically characterized as positive regulators of the immune response, primarily by producing antibodies. However, recent studies indicate that various subsets of B cells can perform regulatory functions mainly through IL-10 secretion. Here we discovered that peritoneal B-1 (B-1P) cells produce high levels of IL-10 upon stimulation with several Toll**-**like receptor (TLR) ligands. High levels of IL-10 suppressed B-1P cell proliferation and differentiation response to all TLR ligands studied in an autocrine manner *in vitro* and *in vivo*. IL-10 that accumulated in cultures inhibited B-1P cells at second and subsequent cell divisions mainly at the G1/S interphase. IL-10 inhibits TLR induced B-1P cell activation by blocking the classical NF-*κ*B pathway. Co-stimulation with CD40 or BAFF abrogated the IL-10 inhibitory effect on B-1P cells during TLR stimulation. Finally, B-1P cells adoptively transferred from the peritoneal cavity of IL-10^−/−^ mice showed better clearance of *Borrelia hermsii* than wild-type B-1P cells. This study described a novel autoregulatory property of B-1P cells mediated by B-1P cell derived IL-10, which may affect the function of B-1P cells in infection and autoimmunity.

## Introduction

Regulation of the immune response is as important as its activation to prevent harmful effects caused by effector cells. Both cell intrinsic (central tolerance) and cell extrinsic (regulatory cells) mechanisms prevent the development of autoimmunity as well as negatively control exaggerated immune responses [1], [2]. Janeway and colleagues were the first to demonstrate a regulatory role for B cells by demonstrating that experimental autoimmune encephalomyelitis (EAE) is enhanced in a B cell deficient environment [3]. B cells that negatively regulate different immune responses through IL-10 production were termed “regulatory B cells” by Mizoguchi and Bhan [4]. Recent studies have shown that IL-10 producing B-cell subsets with varying phenotypes can regulate different immune responses in numerous mouse models, such as inflammatory bowel disease (IBD), EAE, type 1 diabetes, collagen-induced arthritis, contact hypersensitivity and during parasitic infection [5]. Despite the diversity of B cell subsets involved in the disease models, the regulatory mechanisms are uniformly dependent on IL-10 production. One of the high IL-10 producing subsets is the CD1d^hi^CD5^+^ B cell subset, termed “B10 cells” by Yanaba and Tedder [6]. Matsushita et al. showed that depletion of B cells with anti-CD20 antibodies before or during early stages of EAE induction enhanced the disease [7]. B cell depletion during the active disease period decreased the intensity of disease, presumably due to the antigen presenting cell function of B cells. In a clinical trial of B cell depletion therapy for ulcerative colitis, B cell depletion exacerbated the disease [8].

Peritoneal B-1 (B-1P) cells were one of the first B cell subsets to be identified to have the ability to produce IL-10. The B-1 cells were described almost two decades ago and have recently been shown to form a distinct B cell lineage [9]. The B-1 cell subset expresses the pan T cell marker CD5 and is present in the spleen as well as the peritoneal cavity. It is further subdivided into B-1a and B-1b subsets based on differential expression of CD5 versus Mac-1 [10]. The B-1a subset is required for production of natural antibodies whereas the B-1b subset is involved in adaptive immune responses to certain bacterial infections [11], [12], [13]. B-1P cells are the source of natural IgM present in serum, mucosal IgA [10] and play an important role in immunity against bloodborne pathogens [12], [14], [15]. B-1 cells express antibody specificities against conserved bacterial epitopes such as phosphorylcholine as well as self antigens such as ssDNA, Thy1 and red blood cells. In humans, rheumatoid factor producing B cells are present predominantly in the B-1 subset [16]. Also, B-1 cells are elevated in several mouse models of lupus [10]. B-1 cells proliferate poorly in response to BCR crosslinking, presumably to protect against accidental activation by self antigens [17], [18], [19]. This is in part due to negative regulation by CD5 and in part due to defects in generation of synergistic signals via B cell receptor (BCR) and CD19 [17], [20]. Despite the ability of B-1P cells to produce more IL-10 than B-2 cells [21], a regulatory role for them has been shown only in the IBD model [22].

Toll-like receptors (TLRs) are pattern recognition receptors that recognize pathogen associated molecular patterns, which trigger innate immunity leading to initiation of adaptive immunity. Several B cell subsets express TLRs and can be activated via TLR ligands which result in robust proliferation and antibody secretion, even in the absence of dendritic cell activation or aid from T cells [23], [24]. In addition to CD4^+^ T cell help, generation of T-dependent antigen specific antibody responses requires activation of TLRs in B cells [25]. Although this is a controversial finding, it appears to be dependent on the chemical modification of the antigen [26], [27]. TLR signals are also essential for T-independent pathogen-specific IgM response [28]. B-1P cells require intact TLR signaling for the clearance of *Borrelia hermsii*, which causes relapsing fever in both humans and mice [28]. Furthermore, TLR mediated signals synergize with self-antigen mediated BCR signals to stimulate activation of self-reactive B cells [29].

In this study, we demonstrated for the first time that IL-10 plays an auto-regulatory role in B-1P cells. B-1P cells produced much higher levels of IL-10 than all the splenic B cell subsets both constitutively and after stimulation with a variety of TLR ligands. We also made the surprising observation that B-1P cells responded to TLR ligands significantly less than splenic B-2 cells (B-2S) as measured by proliferation and antibody production. High and rapid IL-10 production by B-1P cells upon TLR stimulation inhibited their own proliferation by blocking the classical NF-*κ*B pathway. Co-stimulation with CD40 and BAFF (B cell activating factor belonging to the TNF family), but not IL-5, overcame the IL-10 mediated inhibition of B-1 cells. We showed that high IL-10 production by B-1P cells hampered the clearance of *B. hermsii* in B-1P transferred µMT mice.

## Results

### B-1P cells are hyporesponsive to TLR ligands

**Figure 1:**
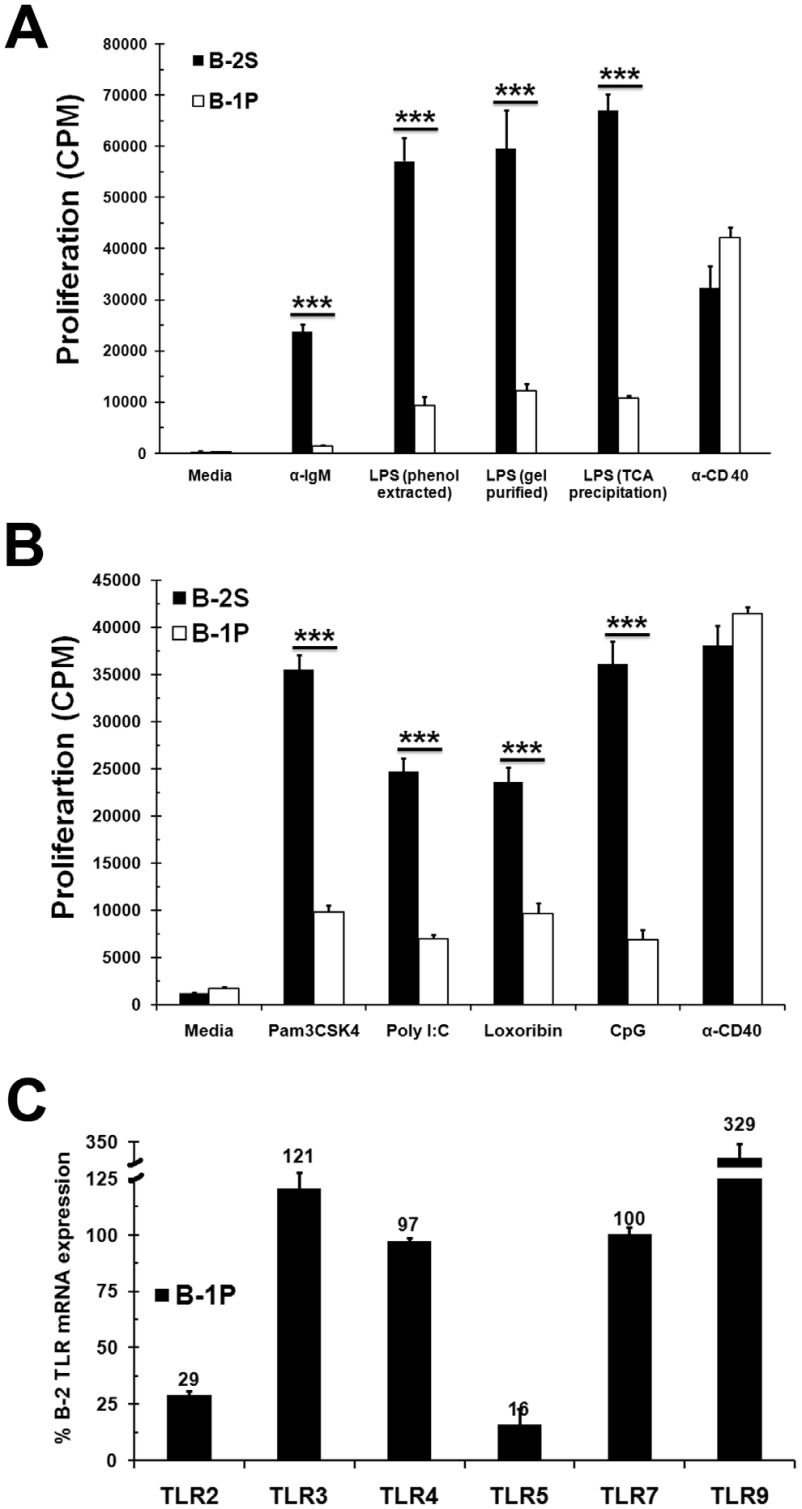
B-1P cells respond weakly to TLR stimulation compared to B-2S cells. (A) B-2S and B-1P cells from C57BL/6 mice were cultured with anti-IgM F(ab')_2_, LPS-2880 (phenol extracted), LPS-2663 (gel purified), LPS-4005 (TCA precipitated) or anti-CD40 for 48 hours and proliferation was measured by ^3^[H] thymidine incorporation. Data are presented as mean ± SD from triplicate cultures and are representative of three independent experiments. (B) B-1P and B-2S cells were cultured with TLR2, 3 and 7 ligands Pam3CSK4, poly I∶C, loxoribine, CpG respectively or anti-CD40 for 48 hours and proliferation was measured by ^3^[H] thymidine incorporation. Similar results were obtained in two other experiments. Data points represent mean ± SD values from triplicate cultures. In panels A and B, ***  = *p*<0.0005 when comparing responses of B-1 and B-2S cells (C) B-1P and B-2S cells were purified and rested for 30 minutes. Total RNA was isolated, and mRNA for various TLRs (TLR-2, 3, 4, 5, 7 and 9) and GAPDH were quantified by quantitative real-time PCR as described under “Experimental Procedures”. Amount of TLR mRNA in B-1P cells was expressed as a percentage of corresponding TLR mRNA in B-2S cells. Data are presented as mean ± SD values from triplicates and are representative of two independent experiments. In all the panels 6-8 mice were used in each experiment.

During the course of our studies to determine if TLR4 and B cell receptor signals synergize in B-1P cells, we made the surprising observation that B-1P cells are hyporesponsive to LPS, a TLR4 ligand, mediated proliferation response when compared to B-2S cells. Both B-1P and B-2S cells responded similarly to CD40 mediated signaling (Fig. 1A). Several previous studies did not emphasize such differences in the LPS response of B-1 and B-2 cells, even though data in some reports supports our observation [30], [31], [32]. The hyporesponsiveness of B-1P cells was independent of the purity (protein or DNA free) of the LPS used (Fig. 1A). On average, we found that the B-1 cell response to LPS is 17±6% of the B-2 cell response (n = 7, *p*<0.004, with an average of 5 mice in each experiment). Interestingly, B-1P cells remained highly viable even up to 6 days after stimulation, while viability of B-2S cells decreased significantly after two days of stimulation, ruling out reduced viability as a cause of B-1P cell hyporesponsiveness to LPS. In spite of this decrease in viability, the absolute number of cells recovered on day 6 was higher in B-2S cells than B-1P cells due to their increased proliferation (Supplementary Fig. S1).

B-1P cell response to other TLR ligands was tested to determine if B-1P hyporesponsiveness was restricted to TLR4 ligand. B-1P cells proliferate significantly less than B-2S cells when stimulated with various TLR agonists, including Pam3CSK4 (TLR-1/2), poly(I∶C) (TLR3), loxoribine (TLR7) and CpG (TLR9). In parallel, both B-1P and B-2S cells proliferated equally well when stimulated via CD40 (Fig. 1B). The TLR5 agonist, flagellin, failed to stimulate B-1P and B-2S cell proliferation. Real time RT-PCR analysis showed that TLR3, TLR4 and TLR7 mRNAs were expressed equally in both B-1P and B-2S subsets, while *TLR2* and *TLR5* genes were expressed less in B-1P than in B-2S cells. Interestingly, TLR9 expression was 3 fold higher in B-1P cells than in B-2S cells (Fig. 1C). Therefore, the hyporesponsivness of B-1P cells to TLR3, 4, 7 and 9 ligands is unlikely to be due to decreased expression of TLR on B-1P cells. We chose LPS and CpG for further studies since LPS uses both TRIF and MyD88 pathways while CpG uses only the MyD88 pathway. They also represent TLRs that differ in cellular expression.

### Hyporesponsiveness of B-1P cells to TLR signaling is in part due to increased production of IL-10

**Figure 2:**
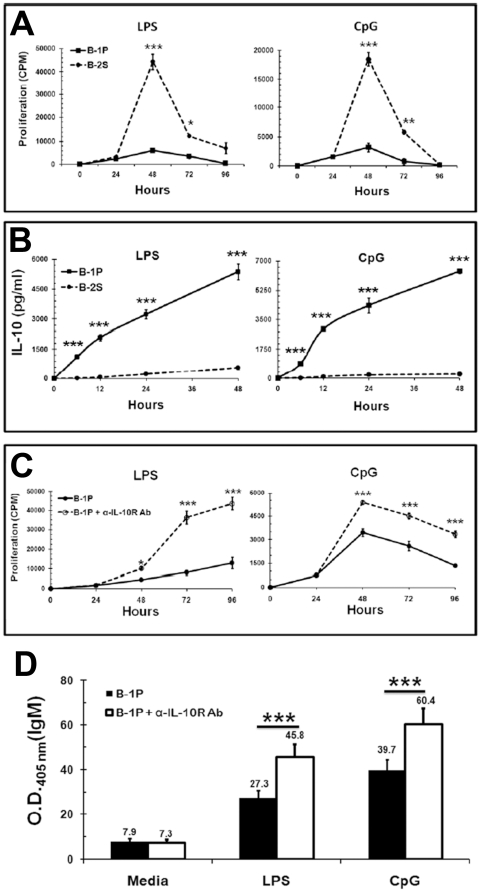
Hyporesponsiveness of B-1P cells to different TLRs is due to high IL-10 production. (A) B-1P and B-2S cells were stimulated with LPS or CpG for 24, 48, 72 and 96 hours in the presence or absence of anti-IL-10R antibody. The cultures were pulsed with ^3^[H] thymidine during the final four hours of the culture period and the results are presented as mean ± SD of triplicate determinations. (B) Culture supernatants of B-2S and B-1P cells were collected at 6, 12, 24 and 48 hours of stimulation with LPS or CpG and assayed by ELISA for IL-10. In panels A and B the *p*-values (* = *p*<0.05, ** = *p*<0.005, *** = *p*<0.0005) denote the significance of differences between the responses of B-1P and B-2S cells. Results presented in panels A and B are representative of two independent experiments with 6–8 mice in each experiment. (C) B-1P cells were cultured with LPS or CpG in the presence or absence of anti-IL-10R antibody or isotype antibody and proliferation was measured by ^3^[H] thymidine incorporation. Data (mean ± SD, n = 10 mice per experiment) are representative of three independent experiments. The *p*-values (* = *p*<0.05, *** = *p*<0.0005) signify the differences between proliferation responses with and without anti-IL-10 antibody. (D) B-1P cells were cultured with LPS or CpG for 5 days in the presence or absence of anti-IL-10R antibody. At the end of 5 days, culture supernatants were collected and assayed by ELISA for total IgM. Results (mean ± SD, n = 6 mice per experiment) are representative of three experiments. The *p*-value (*** = *p*<0.0005) signify differences in antibody secretion with and without anti-IL-10 antibody.

An examination of the time kinetics revealed that B-1P and B-2S cell proliferation responses to LPS and CpG were similar after 24 hours of stimulation. However, B-2S response peaked at 48 hours and remained significantly higher than B-1P cells up to 72 hours (Fig. 2A). This difference in kinetics, which is also dependent on cell densities, may be the reason why many previous studies [19], [30], [31], [32] did not appreciate the difference in TLR response of B-1 and B-2 cells. IL-10 inhibits *in vitro* proliferation of murine B-2S cells and human activated leukemic CD5^+^ B cells [33], [34]. Hence, we hypothesized that hyporesponsiveness of B-1P cells to LPS and CpG may be due to production of IL-10 by B-1 cells and its inhibitory effects. However, the kinetics of IL-10 production by B-1P and B-2S cells upon TLR stimulation has never been examined previously. As shown in Fig. 2B, B-1P cells produced large amounts of IL-10, with quantities detectable as early as 6 hours after LPS or CpG stimulation, while B-2S cells produced very low levels of IL-10, which became detectable only after 12 hours of LPS (TLR4) or CpG (TLR9) stimulation. At 48 hours, B-1P cells produced 10 fold more IL-10 upon LPS stimulation and 25 fold more IL-10 upon CpG stimulation than B-2S cells (Fig. 2B). Upon neutralization of IL-10 effects with anti-IL-10R antibody, there was a significant (*p*<0.0004) increase in LPS and CpG induced proliferation of B-1P cells at 48, 72 and 96 hours, but not at 24 hours (Fig. 2C). There was no significant difference (*p*>0.05) in B-2S cell proliferation with anti-IL-10R antibody treatment (Supplementary Fig. S2). Next, we examined if B-1 cell derived IL-10 also plays a role in their differentiation responses. Neutralization of IL-10 increased antibody production by TLR4 or TLR9 stimulated B-1P cells (Fig. 2D), but no significant difference was observed in the B-2S group with the same treatment (Supplementary Fig. S3).

Agonists for TLR2, 3 and 7 also behave like TLR4 and TLR9 agonists in inducing very high amounts of IL-10 secretion by B-1P cells but not by B-2S cells (Table 1). In terms of absolute magnitude, IL-10 induced by TLR ligands ranged from 4 to 12 ng/ml for B-1P cells whereas it was 0.01 to 0.13 ng/ml for B-2S cells. It is also interesting to note that B-1P cells produced high levels of IL-10 constitutively (Table 1). IL-10 appeared to have a role in the reduced B-1 cell responses to all the TLR ligands studied, since there was a significant (*p*<0.006) increase in proliferation of B-1P cells when stimulated with different TLR agonists in the presence of anti-IL-10R antibody in comparison to stimulation with TLR agonists alone (Table 1).

**Table 1:** TLR induced IL-10 production by B-2S and B-1P cells and its inhibitory effects on B-1P cells.

		B-2S	B-1P		
TLR	Ligand	IL-10 (pg/ml)	IL-10 (pg/ml)	% increase in proliferation with anti-IL-10R Ab	*p*-value
	Media	3	345	-	-
TLR2	Pam3CSK4	43	4187	169	0.0001
TLR3	Poly I∶C	15	8019	166	0.0013
TLR7	Loxoribine	129	11990	154	0.0100

B cell subsets were cultured at 10^5^ cells/well with or without different TLR ligand for 2 days. IL-10 was quantified by ELISA. Proliferation was measured after 48 hours either in the presence or absence of α-IL-10R Ab (1 µg/ml). The tritiated thymidine incorporation for B-1P cells stimulated with Pam3CSK4, Poly I∶C and Loxoribine in the absence of α-IL-10R Ab was 9830, 6989 and 9677, respectively.

### B-1a cells, among different B cell subsets, produce high amounts of IL-10 constitutively and upon TLR stimulation

**Figure 3:**
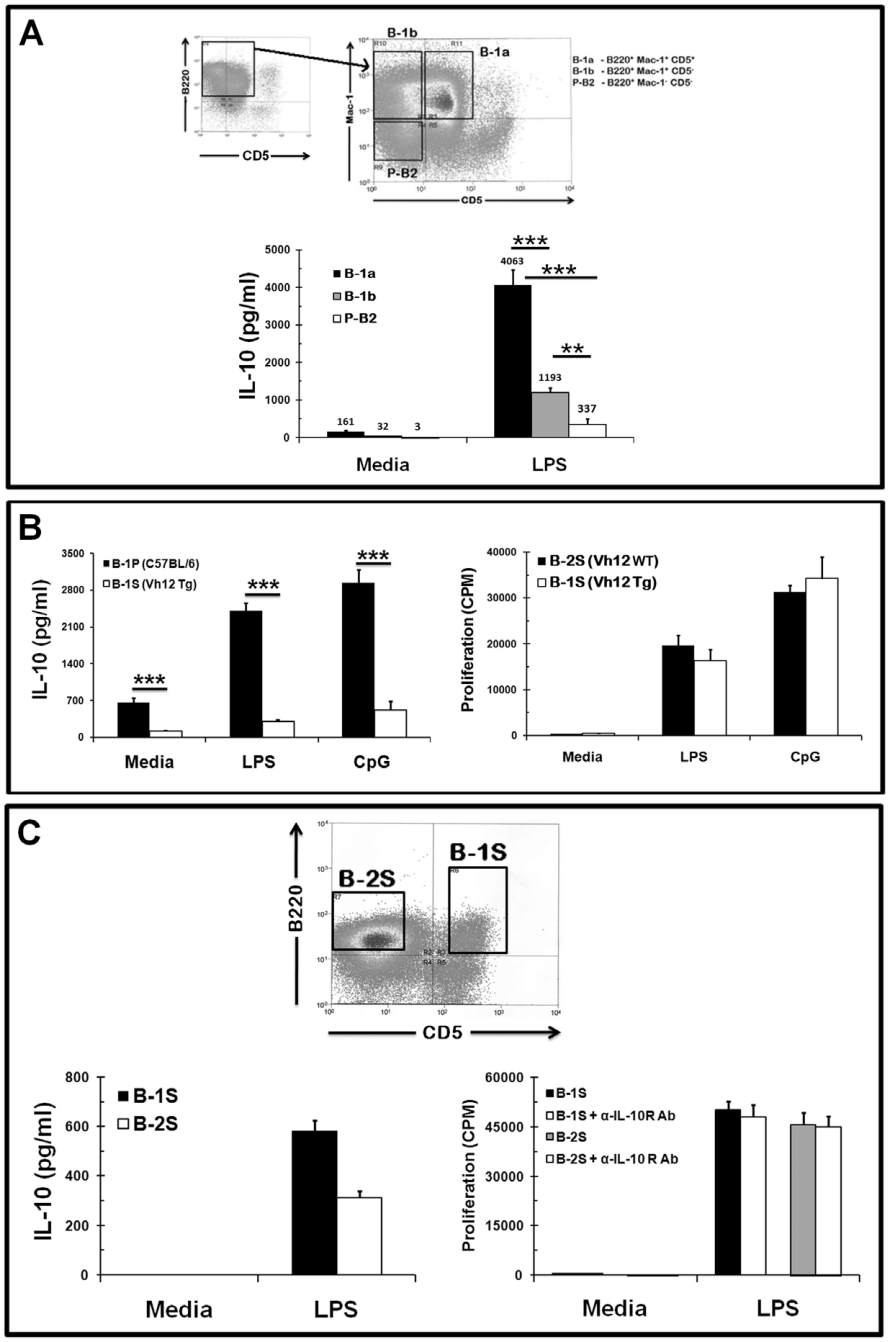
B-1a cells are the major IL-10 producing cells among peritoneal and splenic B cell populations. (A) Macrophage depleted peritoneal cell populations were gated on B220^+^ cells and further separated into B-1a (Mac1^+^CD5^+^), B-1b (Mac1^+^CD5^−^) and B-2 (Mac1^−^CD5^−^) using a FACS machine (top panel). Sorted B-1a, B-1b and B-2 cells were cultured with LPS for 48 hours. The culture supernatants were collected and assayed for IL-10 (bottom panel). Data are shown as mean ± SD of triplicate cultures and are representative of two independent experiments with 15 mice each. The *p*-values signify the differences in IL-10 production by B-1a cells in comparison to B-1b and B-2P cells (*** = *p*<0.0005) and between B-1b and B-2P cells (** = *p*<0.005). (B) B-1P cells from C57BL/6 and B-1S cell from V_H_12 Tg mice were cultured with LPS or CpG for 48 hours; culture supernatants were collected at 48 hours and assayed for IL-10 (left panel). B-2S cells from V_H_12 wild type littermate (WT) mice and B-1S cell from V_H_12 Tg mice were cultured with LPS or CpG for 48 hours and cell proliferation was assessed by ^3^[H] thymidine incorporation (right panel). Similar results were obtained in two other experiments (mean ± SD with five mice per group). The *p*-value (*** = *p*<0.0005) signify the differences in response between B-1P and B-1S cells. (C) Total spleen cells from C57BL/6 mice were sorted into B-2S (B220^+^CD5^−^) and B-1S (B220^+^CD5^+^) cells on the basis of CD5 expression. Sorted B-2S and B-1S cells were cultured with LPS for 48 hours, culture supernatants were collected and assayed for IL-10 (bottom left panel), and for proliferation (bottom right panel) with or without anti-IL-10R antibody. Representative results from the one of two experiments are shown (mean ± SD, n = 5).

B-1P cells can be further divided into B-1a and B-1b cells, both of which express Mac-1 and B220, but only B-1a cells express CD5. It has been shown that CD5 promotes IL-10 production in human B cells [10]. Furthermore, CD5^+^ B lymphoma cells produce increased levels of IL-10 relative to CD5^−^ lymphoma cells [35]. We purified the three peritoneal B cell subsets, B-1a (B220^+^Mac-1^+^CD5^+^), B-1b (B220^+^Mac-1^+^CD5^−^) and B-2P (B220^+^Mac-1^−^CD5^−^) cells, by FACS sorting (Fig. 3A). Among these highly pure subsets (>98%), B-1a cells produced constitutively higher amounts of IL-10 (161 pg/ml) compared to B-1b (32 pg/ml) and B-2P (3 pg/ml) cells. B-1a cells also produced very high levels of IL-10 upon LPS stimulation; almost 12 fold more than B-2P cells and 3.5 fold more than B-1b cells (Fig. 3A, bottom panel). The high amounts of IL-10 produced by B-1a cells inhibited their own proliferation, while the amounts of IL-10 produced by B-1b and B-2P cells (Supplementary Fig. S4) were not sufficient to inhibit their own proliferation. We also FACS-purified the different splenic B cell subsets, specifically the follicular and marginal zone B cells and tested for their ability to produce IL-10 constitutively and after LPS stimulation. As shown in table 2, none of the splenic B cell subsets produced constitutively high levels of IL-10. Similarly, none of the splenic B cell subsets or lymph node B cells produced high amounts of IL-10 upon LPS stimulation in comparison to peritoneal B-1a cells. Although marginal zone B cells produced as much IL-10 as B-1b cells, like the B-1b cells the amount of IL-10 produced was not sufficient to inhibit their own proliferation (Supplementary Fig. S5).

**Table 2:** Constitutive and LPS induced IL-10 production by various murine B cell subsets.

Anatomical location	B cell subset	Surface phenotype	IL-10 (pg/ml)	
			Media	LPS
Peritoneum	B-1a cells	B220^+^Mac-1^+^CD5^+^	161±32	4063±406
	B-1b cells	B220^+^Mac-1^+^CD5^−^	32±9	1193±126
	B-2 cells	B220^+^Mac-1^−^CD5^−^	3±1	337±166
Spleen	B-1 cells	B220^+^Mac-1^−^CD5^+^	ND	583±42
	Marginal Zone B cells	B220^+^AA4.1^−^CD21^hi^ CD23^lo^	9±5	1054±32
	Follicular B cells	B220^+^AA4.1^−^CD21^int^ CD23^hi^	1±2	136±12
	Total B cells	B220^+^	ND	313±26
Mesenteric Lymph Nodes	Total B cells	B220^+^	1±2	240±18

B cell subsets in the peritoneum, spleen and mesenteric lymph node were separated by FACS sorting (≥96% pure) and were cultured at 10^5^ cells/well with or without 5 µg/ml LPS for 2 days. IL-10 was quantified by ELISA. ND  =  non-detectable.

### B-1S cells behave more like B-2S cells, rather than B-1P cells, in terms of IL-10 production and proliferation

Even though the majority of B-1 cells are in the peritoneum, substantial numbers of B-1 cells are also located in the spleen. B-1P cells, unlike splenic B-1 (B-1S) cells, express a constitutively activated form of STAT3, a transcription factor, known to regulate IL-10 gene expression [30]. B-1S cells from V_H_12 transgenic mice (V_H_12 Tg) (Most V_H_12 B cells in adult mice bind the common phospholipid phosphatidylcholine (PtC) and are B-1 [36]) showed very low constitutive IL-10 production and upon LPS or CpG stimulation in comparison to B-1P cells (Fig. 3B, left panel). Furthermore, B-1S cells from V_H_12 Tg mice did not show any hyporesponsiveness to LPS or CpG stimulation in comparison to B-2S cells from V_H_12 wild type littermate (V_H_12 WT) (Fig. 3B, right panel). We could not detect any IL-10 in unstimulated wild type B-1S cells even after FACS sorting (Fig. 3C, top panel), suggesting that both B-2S and B-1S cells lacked the ability to produce IL-10 constitutively. Upon LPS stimulation, both B-1S and B-2S produced comparable amounts of IL-10 (Fig. 3Cbottom left panel, ), but the amount is significantly less than B-1P cells (Fig. 2B & 3A). As expected, the low levels of IL-10 produced by B-1S and B-2S cells did not have an inhibitory effect on their proliferation response to LPS, as shown by a lack of increase in proliferation upon IL-10 neutralization (Fig. 3C, bottom right panel). This data regarding IL-10 production by splenic B-1a cells is in agreement with the identification of IL-10 producing B10 cells, characterized as CD1d^high^CD5^+^ cells, by Yanaba et al. [6]. However, it is unclear if the slpenic CD1d^high^CD5^+^ B10 subset will produce as much IL-10 as the peritoneal B-1a cells, since Yanaba et al. [6] used only intracellular staining to identify B-10 cells.

### IL-10 regulates B-1P response: in vitro and in vivo

**Figure 4:**
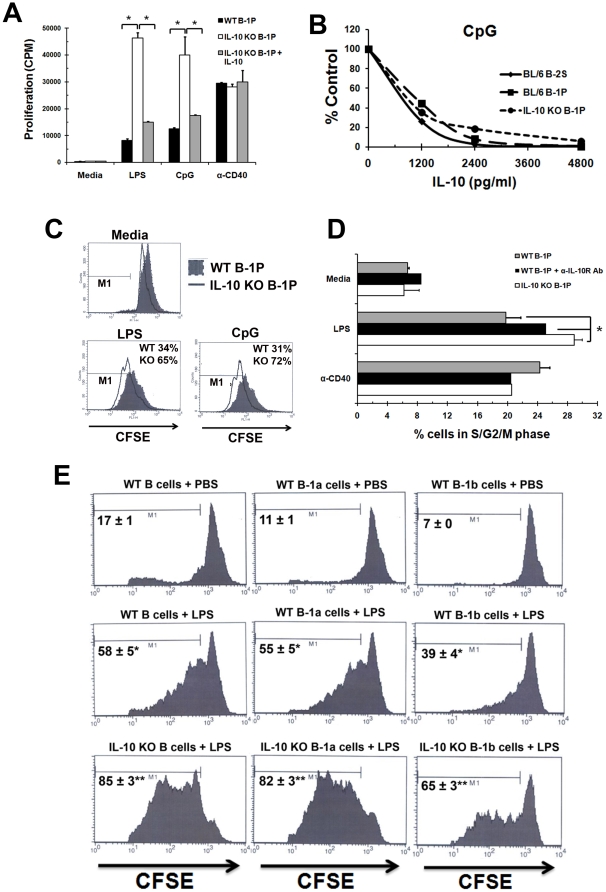
IL-10 inhibits B-1P cell response by arresting the cells in G0/G1 phase of cell cycle. (A) B-1P cells from C57BL/6 and IL-10^−/−^ mice were cultured with LPS, CpG or anti-CD40 for 48 hours. IL-10 (2500 pg/ml) was added to some of the cultures of IL-10^−/−^ B-1P cells. Results (mean ± SD of triplicate cultures) are representative of three experiments with seven mice per experiment. * = *p*<0.05 indicates statistical significance of the response between WT B-1P and IL-10^−/−^ B-1P or also with and without added IL-10. (B) Dose dependence of IL-10 mediated inhibition of B-2S, WT B-1P and IL10^−/−^ B-1P cell proliferation responses to CpG. Similar results were obtained in three other experiments with 4–6 mice per experiment. (C) CFSE labeled B-1P cells (10^5^/well) from C57BL/6 and IL-10^−/−^ mice were cultured with LPS or CpG for 48 hours and were analyzed for CFSE dilution after gating for B220 and CD11b double positive cells. The percentage cells in M1 regional gate represent CFSE dilution from the second and subsequent divisions of cells based on the modfit analysis of CFSE fluorescence (D) B-1P cells from C57BL/6 and IL-10^−/−^ mice were cultured with LPS or anti-CD40 in the presence or absence of anti-IL-10R antibody for 48 hours and cell cycle analysis was performed by PI staining. The fraction of cells in G2/S/M was determined from triplicate cultures and the mean ± SD values are plotted. Similar results were obtained in two other experiments with seven mice per group. The statistical significance of difference in response with or without anti-IL-10R antibody or between wild type and IL-10 knockout B-1P cells is shown by * = *p*<0.005. (E) CFSE labeled purified peritoneal B cells (B220^+^) from wild type or IL-10^−/−^ mice were injected intraperitoneally into Rag-1^−/−^ mice. Two hours later 25 µg of LPS per mouse was injected intraperitoneally. Total B-1P cells (B220^+^) (left panel), B-1a cells (B220^+^Mac-1^hi^) (middle panel) and B-1b cells (B220^+^Mac-1^lo^) (right panel) from wild type or IL-10^−/−^ transferred mice were analyzed for CFSE dilution after 60 Hours of *in vivo* stimulation with PBS or LPS. (* = *p*<0.005, compared to PBS treated group, ** = *p*<0.005, compared to WT B-1 transferred group with LPS stimulation)

Since neutralization of IL-10 signaling via the anti-IL-10R antibody led to an increased response of B-1P to TLR stimulation, we determined if the B-1P cells from IL-10^−/−^ mice were also more responsive to TLR signaling. B-1P cells from young IL-10^−/−^ mice proliferated significantly more upon LPS (>5 fold) and CpG (>3 fold) stimulation when compared to B-1P cells from wild type mice (Fig. 4A). Wild-type and IL-10^−/−^ B-1P cells proliferated equally upon CD40 stimulation. The TLR responses of IL-10^−/−^ B-1P cells were still susceptible to IL-10 inhibition, since exogenously added IL-10 inhibited their response to LPS and CpG (Fig. 4A). LPS (data not shown) or CpG (Fig. 4B) induced B-1P (WT and IL-10^−/−^) and B-2S proliferation was inhibited similarly by exogenous IL-10.

Since there was no difference in proliferation response of B-1 and B-2 cells at 24 hours (Fig. 2A), nor was the 24 hour response enhanced by IL-10 neutralization in B-1P cells (Fig. 2C), we hypothesized that the TLR induced IL-10 mediated inhibition does not affect the first or initial rounds of cell division, as it takes time to accumulate high levels of IL-10. CFSE staining revealed that wild-type B-1P cells have decreased number of cells in the second and subsequent rounds of cell division when compared to IL-10^−/−^ B-1P cells upon LPS or CpG stimulation (Fig. 4C), which supports our hypothesis. A cell cycle analysis by Propidium Iodide (PI) staining showed that upon LPS stimulation, fewer wild-type B-1P cells were present in S/G2/M phase when compared to wild-type B-1P cells stimulated in the presence of anti-IL-10R antibody or in IL-10^−/−^ B-1P cells (Fig. 4D). No significant difference was observed in the B-2S group with the same treatment (Supplementary Fig. S6). Thus, IL-10 appears to arrest B-1 cell division at the G0/G1 stage of the cell cycle.

We further analyzed the IL-10 auto-regulatory effect on B-1P cells *in vivo*. To demonstrate the autocrine and paracrine effects of B-1 cell derived IL-10, we transferred 3×10^6^ CFSE-labeled peritoneal B cells from wild type and IL-10^−/−^ mice into Rag-1^−/−^ mice intraperitoneally. The recipients were challenged with 25 µg/ml LPS. The CFSE-labeled B cells were then isolated from the peritoneal cavity and the CFSE fluorescence of B-1a and B-1b B cells was analyzed. As shown in Fig. 4E total peritoneal B cells as well as B-1a cells from IL-10^−/−^ mice exhibited greater proliferation than same subsets from the wild type mice suggesting that IL-10 produced by B-1a cells can have autocrine inhibitory effects in an *in vivo* situation. Moreover, B-1b cells from IL-10^−/−^ mice also exhibited increased proliferation suggesting that IL-10 from B-1a cells (and/or by themselves) can have paracrine inhibitory effects.

### Alternate NF-κB signaling, but not JAK/STAT signaling, can overcome inhibitory effects of IL-10 on B-1P cells

**Figure 5:**
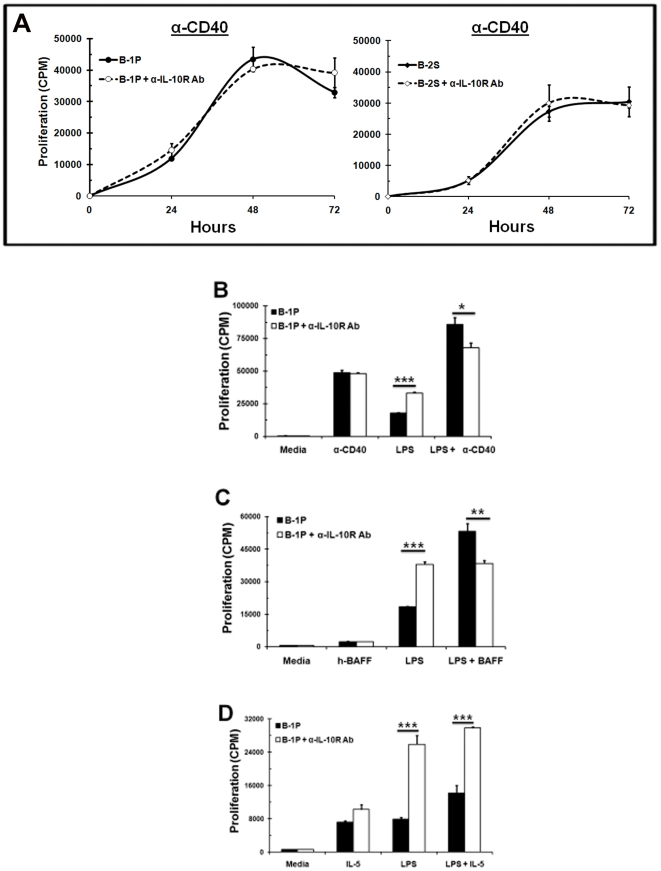
CD40 and BAFF, but not IL-5, overcome the inhibitory effects of IL-10. B-2S and B-1P cells were cultured with (A) α-CD40 (B) LPS, anti-CD40 or both together (C) LPS, BAFF or both together (D) LPS, IL-5 (10 ng/ml) or both together for 48 hours in the presence or absence of anti-IL-10R antibody. Cell proliferation was measured by ^3^[H] thymidine incorporation. Results (shown as mean ± SD responses of triplicate cultures) are representative of three experiments with 8 mice per experiment. The *p*-values (* = *p*<0.05, ** = *p*<0.005, *** = *p*<0.0005) depict significance of difference in the proliferation response with and without anti-IL-10R antibody.

Although CD40 stimulation induced IL-10 production (950 pg/ml) by B-1P cells, the levels were much lower in comparison to TLR4 stimulation (5380 pg/ml). No noticeable differences were found in CD40 induced proliferation of B-1P and B-2S cells (Fig. 1A & Fig. 1B). Moreover, neutralization of IL-10 signals with anti-IL-10R antibody did not change the proliferation response of either B-1P or B-2S cells to anti-CD40 (Fig. 5A). Hence, we examined if CD40 co-stimulation can overcome the IL-10 mediated inhibition of TLR responses in B-1P cells.

CD40 co-stimulation with LPS resulted in a proliferation response that was more than additive, which was not further enhanced upon neutralization of IL-10 signals, but was reduced slightly (Fig. 5B). CD40 signals via both alternate and classical NF-*κ*B pathways, whereas TLRs signal via the classical NF-*κ*B pathway [37], [38]. In monocytes, exogenous IL-10 inhibits TLR signaling by blocking classical NF-*κ*B pathways via inhibition of IκB kinase (IKK) activity or p50/p65 translocation to nucleus, or by selectively inducing nuclear translocation of p50/p50 homodimer [39], [40], [41]. Hence, absence of inhibition of CD40 responses could be due to the activation of alternate NF-*κ*B pathway. BAFF, which is critical for survival and maturation of B cells, also signals via the alternate NF-*κ*B pathway [38]. Therefore, we examined if BAFF co-stimulation can overcome the IL-10 mediated inhibition in B-1 cells. BAFF co-stimulation with LPS leads to a synergistic proliferation response, which was not enhanced by IL-10 neutralization (Fig. 5C). Instead we found a slight decrease in proliferation response of B-1P cells upon LPS co-stimulation with anti-CD40 or BAFF in the presence of anti-IL-10R antibody. It is conceivable that in the presence of CD40 and BAFF, two agents known to stimulate alternate NF-κB signaling, IL-10 has a prosurvival effect on B cells.

B-1 cells express the IL-5 receptor and IL-5 acts as a survival and proliferation factor for B-1 cells by signaling via JAK/STAT pathway [42]. IL-5 co-stimulation with LPS resulted in an increase in proliferation response which was significantly enhanced when IL-10 signaling was blocked (Fig. 5D). Similar results were obtained with IL-2 and IL-4 (data not shown). Thus, co-stimulation via alternate NF-*κ*B signaling (CD40, BAFF), but not JAK/STAT signaling (IL-2, IL-4, IL-5), can overcome the IL-10 mediated inhibition of B-1P cells upon TLR stimulation.

### IL-10 inhibits NF-κB nuclear translocation by preventing LPS-induced degradation of IκBα

**Figure 6:**
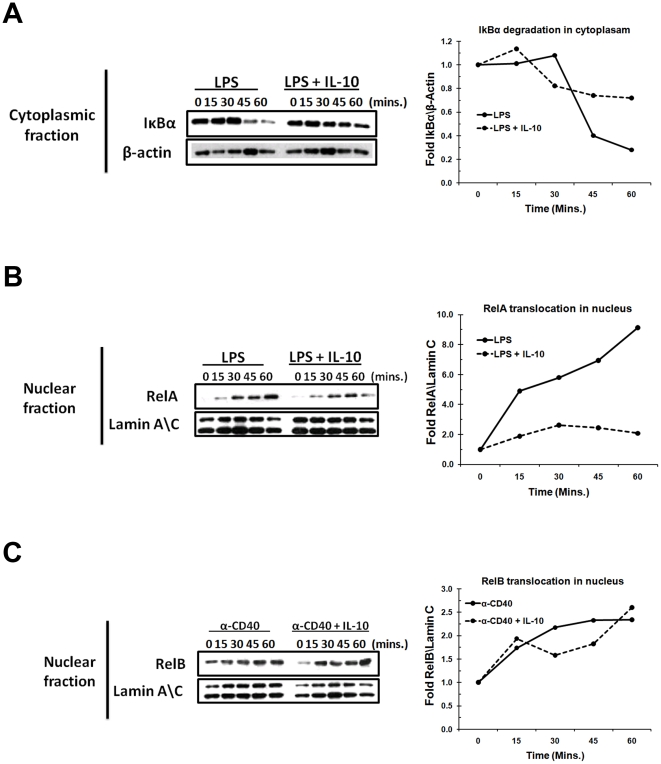
Effect of IL-10 on LPS and CD40 induced NF-κB activation in B-2S cells. Lysates from B-2 S cells at different time points were analyzed by western blot technique as described under “materials and methods.” (A) IκBα protein expression in cytoplasmic fractions of LPS-stimulated cells. (B) RelA protein expression in nuclear fractions of LPS-stimulated cells. (C) RelB protein expression in nuclear fractions of CD40-stimulated cells. β-actin and lamin A\C were used as a control for cytoplasmic and nuclear fractions, respectively. Fold difference in the protein expression are represented in line graphs after normalization with loading controls.

Since IL-10 inhibited LPS (known to activate NF-*κ*B by the classical pathway) but not CD40 (known to activate NF-κB by both classical and non-classical pathways) induced B-cell growth responses, we tested if IL-10 has differential effects on the two pathways of NF-κB activation. We used B-2 cells for these studies because both B-2 and B-1 cells are similarly susceptible to IL-10 mediated inhibition when stimulated with TLRs (Fig. 4B). B-2S cells were stimulated with LPS for indicated time points in the presence or absence of IL-10 (as described in Methods). As shown in Fig. 6A, LPS stimulation resulted in a time-dependent degradation of IκBα. In contrast, pretreatment of IL-10 decreased IκBα degradation. To further elucidate if IL-10 induced blocking of IκBα degradation inhibits RelA (p65) translocation into nucleus, we examined the accumulation of RelA in the nuclear fraction of LPS stimulated cells in the presence or absence of IL-10. LPS stimulated cells showed increased nuclear translocation of RelA compared to IL-10 pretreated cells (Fig. 6B). There was up to a 9 fold increase in nuclear translocation of RelA upon LPS stimulation in the absence of IL-10. However, there was only a 2.5 fold increase in nuclear translocation of RelA when cells were pre-incubated with IL-10 (Fig. 6B).

As noted above, CD40 signaling can activate NF-κB by the alternate pathway, which involves translocation of RelB into the nucleus. We confirmed this by showing that RelB is found in increased amounts in the nuclear lysates of CD40-activated B cells (Fig. 6C). Treatment with IL-10 did not affect nuclear levels of RelB in CD40 stimulated B cells at most time points examined. At two time points (30′ and 45′) there was a small decrease in nuclear levels of RelB in IL-10 treated cells which was not sustained at the 60′ time point. These results all together indicate that pretreatement with IL-10 blocks LPS induced classical NF-κB pathway, via IκBα degradation and RelA translocation to nucleus, but not the CD40 induced alternate NF-κB pathway.

### Production of IL-10 by B-1P cells limits clearance of B. hermsii from the blood

B-1P cells play a primary role in the clearance of *B. hermsii* [12]. TLR2 plays a major role in the activation of B cells when they come in contact with *B. hermsii* lipoproteins and leads to B cell proliferation and differentiation to produce antibodies against *B. hermsii* lipoproteins [43]. Even though, TLR2 signaling plays a major role in the activation of B-1 cells, it is not the only TLR required for response against *B. hermsii*, as MyD88 knockout mice suffer from more severe episodes of bacteremia with *B. hermsii* than TLR2 knockout mice [28].

**Figure 7:**
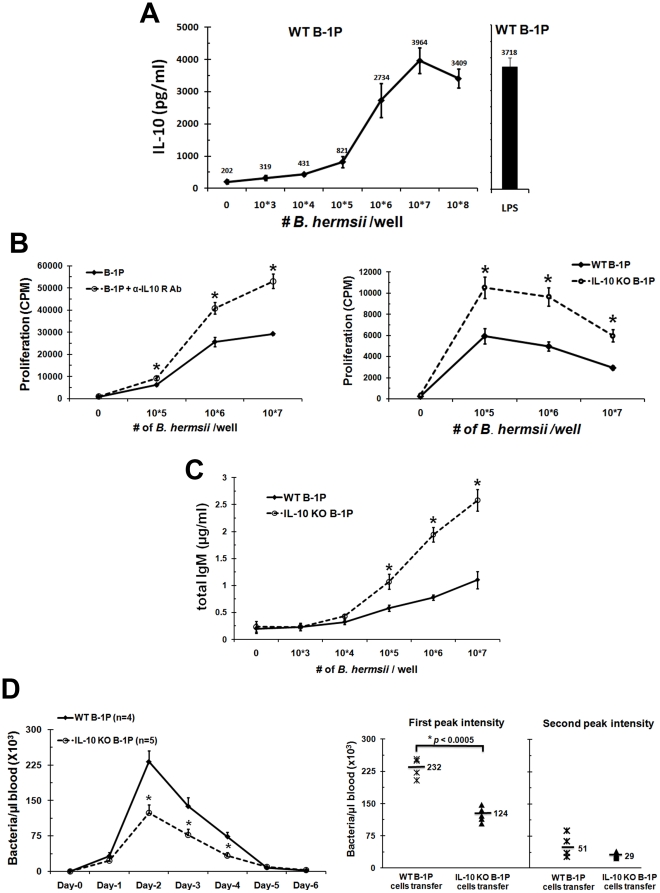
Role of IL-10 in B-1P cell responses to *B. hermsii* in vitro and in vivo. (A) B-1P cells from C57BL/6 mice were cultured with different numbers of live *B. hermsii* or LPS for 48 hours and culture supernatants were analyzed for IL-10 secretion by ELISA. (B) Wild type B-1P cells in the presence or absence of α-IL-10R antibody (left panel) or B-1P cells from C57BL/6 and IL-10^−/−^ mice (right panel) were cultured with different numbers of live *B. hermsii* for 48 hours and proliferation was measured by ^3^[H] thymidine incorporation. Representative results (mean ± SD) from the one of three experiments are shown (seven mice per group in each experiment). The statistical significance of difference in response with or without anti-IL-10R antibody or between wild type and IL-10 knockout B-1P cells is shown by * = *p*<0.05. (C) B-1P cells from C57BL/6 and IL-10^−/−^ mice were cultured with different numbers of live *B. hermsii* for 5 days and total IgM production was measured by ELISA. (D) µMT mice transferred with 2×10^6^ wild-type (n = 4) or IL-10^−/−^ B-1P (n = 5) cells were infected with *B. hermsii* and bacterial numbers in the blood were quantified by dark field microscopy every 24 hours (left panel). Mean values of *B. hermsii* numbers in each group during the first and second peak (day-10) of bacteremia are shown in the right panel. Results for the studies in panels B, C and D are from one of two experiments, both of which had similar outcome. For panel C and D *p*-value (* = *p*<0.05) depict significance of difference in response between WT and IL-10 knockout B-1P cells.

Accordingly, we found that B-1P cells produce high levels of IL-10 when stimulated with *B. hermsii* (Fig. 7A), as we have shown previously with a synthetic TLR2 ligand (Table 1). We also found that *B. hermsii* induced greater proliferation of wild type B-1P cells upon blocking of IL-10 signaling with α-IL-10R antibody (Fig. 7B, left panel). Similarly, B-1P cells from the IL-10^−/−^ mice proliferated better than wild type mice, presumably via a *B. hermsii* associated TLR2 (Fig. 7B, right panel). There is also an increase in antibody production by IL-10^−/−^ B-1P cells compared to WT B-1P cells upon *B. hermsii* stimulation (Fig. 7C).

Therefore, the ability of B-1 cells to produce antibody and clear the *B. hermsii* infection might become restricted due to high levels of IL-10 produced by B-1 cells. To test this hypothesis, we adoptively transferred purified B-1P cells from wild-type or IL-10^−/−^ mice into cohorts of µMT mice (B cell deficient mice). The µMT mice cannot eliminate *B. hermsii* from their blood [44]. The control untransferred µMT mice maintained 178×10^3^ bacteria/µl of blood up to 10 days without any relapse. The B-1 cells provided by adoptive transfer were sufficient to control *B. hermsii*, as both groups of adoptively-transferred mice achieved peak bacteremia at day 2 and cleared bacteria by day 5 or 6. Interestingly, levels of *B. hermsii* in the blood of mice which received wild-type B-1P cells were significantly higher than in the mice that received IL-10^−/−^ B-1P cells (days 2 to 4, Fig. 7D). The first bacteremic episode peaked at bacterial densities that were nearly twice as high in mice that received wild-type B-1P than in mice that received IL-10^−/−^ B-1P cells (*p*<0.0005; Fig. 7D). Although there was some mouse to mouse variation, there were no significant differences in the persistence of the first bacteremia episode of either group. The second bacteremia peak also trended toward higher bacterial densities in the wild-type B-1 cell transferred group than in the IL-10^−/−^ B-1 cell transferred group, but the differences were not statistically significant (*p*<0.11; Fig.7D). These results indicate that B-1 cell derived IL-10 plays a significant role during *B. hermsii* infection and impairs the ability of B-1 cells to clear *B. hermsii* from the blood.

## Discussion

Studies presented here identified the novel property of autoregulation in the B-1 B cell subset, wherein IL-10 produced by B-1 cells inhibits their own functional responses. We have demonstrated that the TLR induced response of B-1P cells is less than that of B-2S cells due to rapid induction of IL-10, which in turn suppresses the proliferation and differentiation of B-1P cells. We used both the anti-IL-10R antibody and IL-10^−/−^ B-1P cells to demonstrate the inhibitory effects of IL-10 on TLR responses of B-1P cells. The susceptibility to inhibition by IL-10 was not unique to the B-1 subset, as similar effects were observed in all studied B cell subsets once adequate levels of IL-10 were present. B-1P cells were unique in that they produced very high levels of IL-10 leading to a feedback inhibition of their TLR responses and hence we call them “autoregulatory B cells”. This autoregulatory property of B-1P cells appears to have a physiological affect in moderating the B-1 cell responses to bacterial infection. The inhibitory effect of high doses of IL-10 on B cell proliferation is consistent with previously reported observations that high levels of exogenous IL-10 inhibit proliferation of human leukemic CD5^+^ B-cells [33], superantigen induced T cells [45] and macrophage proliferation [46]. The IL-10 mediated autoregulation is not restricted to TLR responses, since similar effects were also observed in B-1P cell response to BCR cross-linking (manuscript in preparation).

The rapid and high levels of IL-10 production were observed with all TLR ligands studied except flagellin, a TLR5 ligand, which did not induce a measurable proliferation response. The B-1 cell proliferation response to all the TLR ligands was less than that of the B-2 cell response and in all cases could be enhanced by neutralization of the inhibitory effects of IL-10. Thus, the IL-10 induction by TLR ligands was seen with both MyD88 dependent and independent stimuli. This may relate to the previously described constitutive expression of STAT-3 by B-1 cells, a transcription factor required for IL-10 production [30]. The transcription factors Sp3, c-maf, IRF, CREB and NF-*κ*B have a role in IL-10 transcription [47]. Moreover, IL-10 has also been shown to be regulated at the level of mRNA stability and translation. Future studies will have to determine if any of these factors are also upregulated in the B-1P cell subset to account for their high IL-10 phenotype. Preliminary studies suggest that activated p38MAPkinase may have some role in this, since p38MAPK inhibitors partially mimic the anti-IL-10R antibody treatment in enhancing B-1P cell functional response (Sindhava et al. unpublished data).

The production of high levels of IL-10 both constitutively and after TLR signaling was uniquely associated with the B-1a cell subset and to some extent with the B-1b B cell subset in the peritoneum. B-1a cells showed constitutive IL-10 production, 53 fold higher than peritoneal B-2 and 5 fold higher than peritoneal B-1b cells. Upon LPS stimulation, B-1a cells showed the highest IL-10 production among the peritoneal B cell subsets. This high IL-10 production by B-1a cells also inhibits their own proliferation in an autocrine manner, whereas lower levels of IL-10 secretion by B-1b cells is not enough to inhibit their own proliferation upon LPS stimulation. However, in vivo, the high levels of IL-10 produced by B-1a cells in the peritoneal cavity are capable of suppressing the B-1b B cell responses as demonstrated by our adoptive transfer experiment (Fig. 4E). Thus the IL-10 produced by B-1a cells can have both autocrine and local paracrine effects.

Initially, V_H_12 Tg mice were used as a source of B-1 cells from the spleen. B-1S cells from the mice produced 8-fold less IL-10 in comparison to B-1P cells. Sort-purified B-1S (B220^+^CD5^+^) cells from wild-type mice spleens also behaved like V_H_12 Tg B-1S cells. Accordingly, there was no significant difference in proliferation between V_H_12 Tg B-1S, wild type B-1S and wild type littermate B-2S cells upon LPS stimulation. Additionally, the proliferation of B-1S cells is not affected by the low levels of IL-10 secreted by these cells. Together these results demonstrate that B-1S cells behave more like a B-2S cell, rather than B-1P cells, in terms of IL-10 production and proliferation.

These results do not contradict the identification of IL-10 producing B10 cells in spleen by Yanaba et al. [6]. We do find IL-10 production by Splenic B-1 cells but quantitatively it is less than that induced by peritoneal B-1a cells. Since the B-1S cells from wild-type mice or V_H_12 Tg mice do not exhibit high IL-10 secretion, it is likely that the peritoneal environment has a unique role in the ability of B-1P cells to produce IL-10. Previously, Chumely et al. as well as Stoermann et al. have described the existence of factors in the peritoneum that affect the special characteristics of B-1P cells [48], [49]. The above described constitutive STAT-3 expression may also have a role in IL-10 production by B-1P cells, although STAT-3 expression has been found to be an intrinsic but not an induced property of B-1P cells [30]. Since B1P cells have a major role in mucosal IgA production, it will be interesting to determine if B cells from the mucosal sites also share the property of high IL-10 production with B-1P cells and how they escape the inhibitory effects of IL-10 to produce large quantities of IgA.

Among B cell subsets, marginal zone B cells and transitional 2 B cells, as well as the recently described splenic B10 cell subset, have all been reported to produce IL-10 [5], [6]. The B10 subset expresses CD5 and high levels of CD1d, but CD1d expression is not required for IL-10 production as B-1P cells do not express CD1d [50]. Most of the previous studies used RT-PCR or intracytoplasmic staining, techniques which do not allow for accurate quantification of the secreted IL-10 and thus were unable to appreciate the significant differences between the levels of IL-10 produced by the various B cell subsets. In a side by side comparison, our studies found that the peritoneal B-1a subset produced more IL-10 than follicular B cells, marginal zone B cells and B-1S cells. Not only do B-1P cells produce high levels of IL-10, but they also exhibit accelerated kinetics, which allows for rapid accumulation of IL-10 in cultures and leading to inhibition of the TLR response.

The effect of IL-10 is mainly on the activation of NF-*κ*B by the classical pathway, since IL-10 inhibited the nuclear translocation of LPS-induced p65 but not of CD40-induced p52. This explains why stimuli such as CD40 and BAFF that can signal via the alternate NF-*κ*B pathway are able to overcome the inhibitory effects of IL-10 on TLR responses. Future studies will determine if IL-10 inhibits the upstream NIK, but not the MEKK3, that are pivotal points that distinguish classical and alternate pathways of NF-*κ*B activation.

Several studies have demonstrated that IL-10 can decrease the antimicrobial activities of immune cells. There are also reports that mice in which IL-10 affect has been neutralized show enhanced abilities to clear pathogens such as *Streptococcus*, *Listeria* and *Mycobacterium* species, when compared to mice with functionally active IL-10 [51]. In many of these studies, IL-10 has been shown to affect several cell types, including CD4^+^ T cells and macrophages. Clearance of the relapsing fever causing bacterium, *B. hermsii,* is dependent upon B-1P cells: mice deficient in TLR1, TLR2, or the TLR adaptor protein MyD88 generated anti-*B. hermsii* IgM with delayed kinetics and suffered more severe episodes of bacteremia [28]. The present study found that IL-10^−/−^ B-1P cells proliferated to significantly higher levels than did wild-type B-1P cells upon *B. hermsii* exposure *in vitro*, which is due to autoregulation by induced IL-10 production by wild type B-1P cells. Lazarus et al. observed that IL-10^−/−^ mice are more effective at controlling *B. burgdorferi*, the Lyme disease agent, which was attributed to a lack of IL-10 suppression of non-B-cells [52]. Here we showed that IL-10^−/−^ B-1P cells were significantly better than wild-type B-1P cells in controlling *B. hermsii* infection of µMT mice. Although the effects of IL-10 on myeloid cells may also be important, we also have to consider the direct impact of IL-10 on B cell production of antibodies. In our adoptive transfer system, B-1P cells were the only cells that differed in their ability to produce IL-10 as the non B cells in the host were competent to produce IL-10. B-1b cells have been shown to be more important than B-1a cells in antibody production against *Borrelia* species [12]. Since we used unseparated B-1 cells from wild-type and IL-10 deficient mice, it is conceivable that in vivo B-1b cells also produce high levels of IL-10. Alternatively, large quantities of IL-10 produced by peritoneal B-1a cells inhibit anti-*Borrelia* antibody production by the B-1b cells, which are also present in the peritoneal cavity, as demonstrated by our Rag-1^−/−^ transfer experiment shown in Fig. 4E. We will have to evaluate this phenomenon in infections with other bacteria such as *Streptococcus pneumoniae* where B-1a cells produce the innate antibody response and the B-1b cells produce the adoptive antibody response [11].

B-1 cells have been known to secrete antibodies to single stranded DNA, red blood cell surface molecules and several other self antigens. In the rheumatoid factor transgenic mouse model, Marshak-Rothstein and colleagues have shown that TLR9 signaling via DNA from dying cells is a critical factor for activation of self-reactive B cells [29]. Also, BAFF and MyD88 signaling has been shown to promote lupus like disease independent of T cells [53]. Here we showed that BAFF can overcome IL-10 induced down regulation of B-1 cell responses. Excess TLR activation as seen in Yaa mice with TLR7 duplication or TLR7 transgenic mice also leads to autoimmunity [54].

Conceivably, the autoregulatory properties of B-1 cells described here may play a role in healthy individuals in preventing excessive activation of self-reactive B cells via TLR stimulation.

## Materials and Methods

### Mice and reagents

C57BL/6 mice were obtained from Harlan (Indianapolis, IN, USA). IL-10^−/−^, Rag-1^−/−^ and µMT mice were obtained from The Jackson Laboratory (Bar Harbor, ME, USA). V_H_12 Tg were provided by Dr. Stephen Clarke (University of North Carolina, Chapel Hill, NC) [36]. Mice were housed under specific pathogen-free conditions in micro-isolator cages under the Institutional Animal Care and Use Committee **(**IACUC**)** approved protocol. The University of Kentucky IACUC protocol number for this study is 00680M2004. The described studies are approved under this protocol.

TLR agonists: LPS, CpG and Pam3CSK4 were obtained from Sigma Chemical Co. (St. Louis, MO, USA), UCDNA (Calgary, AB, Canada) and Calbiochem (San Diego, CA, USA), respectively. Loxoribine, poly I∶C and flagellin were obtained from Invivogen (San Diego, CA, USA). The polyclonal goat anti-IgM F(ab')_2_ was obtained from ICN/MP Biomedicals, (Irvine, CA, USA), and the anti-IL-10 receptor (anti-IL-10R) (Clone 1B1.3a) was obtained from BD Biosciences (San Diego, CA, USA). Anti-CD40 (1C10 clone) was a gift from Dr. Maureen Howard and was used as ascites fluid. Anti-RelA, anti-IκBα and anti-β-actin antibodies were obtained from Santa Cruz Biotechnology (Santa Cruz, CA, USA), anti-RelB was obtained from Cell Signaling (Danvers, MA, USA) and anti-lamin A\C was obtained from Upstate (Temecula, CA, USA).

### Purification of B cells

Peritoneal cells were obtained from 2–3 month old C57BL/6 mice by peritoneal lavage with Hank's buffered salt solution. Peritoneal and splenic B cells were purified as described in a previous study [20]. Anti-B220/CD45RB∼APC, anti-CD11b∼PE, anti-CD5∼FITC, anti-CD21∼FITC, anti-CD23∼PE or anti-AA4.1∼APC (BD Pharmingen, San Jose, CA) were used to identify and sort B-1a, B-1b, B-2, marginal zone B and follicular B cells from the peritoneum or spleen of C57BL/6 mice using a MoFlo cytometer (DakoCytomation) [55]. V_H_12 Tg mice and wild-type C57BL/6 mice were used for splenic B-1 (B-1S) cell purification.

### In vitro cell proliferation assay and cytokine analysis

Splenic B-2 (B-2S), B-1S and\or B-1P cells (1−2.5×10^5^) were stimulated with 50 µg/ml polyclonal goat anti-IgM F(ab')_2_, 5 µg/ml LPS, 5 µg/ml CpG, 5 µg/ml Pam3CSK4, 50 µg/ml poly I∶C, 50 µg/ml loxoribine, 50 µg/ml flagellin or 1∶1000 dilution of anti-CD40 (1C10) ascites in the presence or absence of anti-IL-10R antibody (1 µg/ml). Cultures were pulsed with^ 3^[H] thymidine for 4 hours on days 1, 2, 3 or 4, and then harvested (Packard, Meriden, CT); the incorporated radioactivity was then measured using a Matrix 96 β-counter (Packard, Downers Grove, IL). Results are represented as mean ± SD of triplicate cultures.

For cytokine analysis B-1P, B-1S and\or B-2S (1−2.5×10^5^) cells were cultured in triplicate for indicated time points with various stimulants. IL-10 levels in the supernatants were estimated using ELISA with OptEIA kits (PharMingen, San Diego, CA, USA). Results are presented as mean ± SD of triplicate measurements for triplicate cultures. Statistical significance of differences in response was evaluated by unpaired Student's t-test.

### CFSE labeling and Cell Cycle Assays

B cells (resuspended at 10^7^/ml in PBS/0.1% BSA, 10 µM CFSE) were incubated at 37°C for 20 min, and then washed with IF-12 medium +10% FBS as described by Lyons et al. [56]. CFSE-labeled B cells (2.5×10^5^) were cultured in the presence of LPS (5 µg/ml) or CpG (5 µg/ml) for 2 days at 37°C with 5% CO_2_.

B-1P and B-2S cells (2.5×10^5^) were cultured with 5 µg/ml LPS or 1∶1000 dilution of anti-CD40 for 2 days. Cells were fixed in 70% (v/v) ethanol for at least 1 h at 4°C, after which the cells were incubated in a mixture of 1 µg/ml propidium iodine (PI) (Sigma-Aldrich) and 25 µg/ml RNase A (Sigma-Aldrich) at 37°C for at least 30 min. The level of PI fluorescence was measured with a MoFlo flow cytometer. Cell populations at subG1, G1, S, G2/M phase were calculated using the program ModFit.

### Quantitative Real-time PCR

Total RNA was isolated from Purified B-1P and B-2S cells (5×10^6^) with the RNA miniprep (Invitrogen, Carlsbad, CA). RNA was quantified at OD260 using a Beckman DU 530 Life Science UV Spectrophotometer (Beckman Coulter Inc, CA) and 2 µg of total RNA was subsequently used to make cDNA using the Superscript II reverse transcriptase (Invitrogen Corp., Carlsbad, CA) according to the manufacturer's protocol. RT-PCR was performed on an ABI Prism 7000 system (Applied Biosystems, Foster City, CA). Primers for murine TLRs were used as reported previously [57]. The GAPDH-specific primers were used for loading control (IDT technologies, Corallevielle, IA).

### Peritoneal cell transfer and LPS administration

A total of 3×10^6^ CFSE-labeled wild type or IL-10^−\−^ peritoneal B cells were intraperitoneally injected into Rag-1^−/−^ mice. Rag-1^−/−^ mice transferred with wild type peritoneal B cells or IL-10^−\−^ peritoneal B cells were intraperitoneally injected with either 250 µl of PBS (n = 4) or 250 µL of LPS (100 µg/ml) (n = 4) 2 hours after B cell-administration. Total peritoneal cells were recovered from each mouse after 60 hours of PBS or LPS injection and subjected to flow cytometry.

### Western blot analysis

B-2S cells (10×10^6^), pre-incubated with or without IL-10 (5 ng/ml, 12 hours), were stimulated with LPS (5 µg/ml) or anti-CD40 (1∶1000) ascites for the indicated time. Cell pellets were lysed in a nuclear and cytoplasmic extraction reagent (Thermo scientific), proteins separated by SDS-PAGE and processed for western blotting. The blots were developed with HyGLO chemiluminescence substrate (Denville scientific), exposed to Kodak X-Omat films and analyzed by an Eastman Kodak Image Station 2000RT. For re-probing, membranes were stripped using a solution containing 62.5 mM Tris-HCl, 2% SDS, and 100 mM â-mercaptoethanol at 50°C for 30 min.

### Infection and quantification of *B. hermsii* in mice

*B. hermsii* isolate DAH was obtained from Dr. Tom Schwan (Rocky Mountain Laboratories, NIH, Hamilton, MT, USA). Efficient experimental *B. hermsii* infection is best achieved using host-adapted spirochetes, i.e. bacteria taken directly from the blood of other infected mice [44], [58]. 8–12 week old C57BL/6 wild-type mice (“donors”) were infected by intraperitoneal injection with 5×10^5^ *B. hermsii* DAH from a mid-exponential phase culture. At the peak of the first bacteremia, the donor mice were euthanized and exsanguinated. Donor mouse blood in citrate buffer was pooled and bacterial concentration determined by darkfield microscopy using a Petroff-Hausser counting chamber.

Two cohorts of B cell-deficient (µMT) mice were adoptively transferred with B-1P cells (2×10^6^ cells/mouse) purified from either wild-type or IL-10^−/−^ mice. One week after transfer, the mice were infected by intraperitoneal injection of 3×10^5^ *B. hermsii* harvested freshly from donor mice. µMT mice that had not undergone adoptive transfer were likewise infected with *B. hermsii*, as a control. Densities of *B. hermsii* in infected mouse blood were quantified every 24 hours as described by Alugupalli et al. [12].

### Statistical analysis

Paired student's *t*-test was used to determine statistical significance of differences between various groups.

## Supporting Information

Figure S1 B2S and B1P were plated at a cell density of 10e5 cells/well and stimulated with LPS (5 µg/ml). Cell viability was measured at days -2, 4 and 6 by the trypan blue dye exclusion method.
(0.19 MB TIF)

Figure S2 B-2S cells (10e5 cells/well) were cultured with LPS (5 µg/ml) in the presence or absence of anti-IL-10R antibody (1 µg/ml); proliferation was measured by 3[H] thymidine incorporation.
(0.14 MB TIF)

Figure S3 B-2S cells (10e5 cells/well) were cultured with LPS (5 µg/ml) or CpG (5 µg/ml) for 5 days in the presence or absence of anti-IL-10R antibody (1 µg/ml). At the end of 5 days culture supernatants were collected and assayed by ELISA for total IgM.
(0.29 MB TIF)

Figure S4 FACS sorted peritoneal B-1a, B-1b and B-2P cells (10e5 cells/well) were cultured with LPS (5 µg/ml) in the presence or absence of anti-IL-10R antibody (1 µg/ml) for 48 hours. Cell proliferation was determined by 3[H] thymidine incorporation.
(0.32 MB TIF)

Figure S5 FACS sorted splenic marginal zone (MZ) and follicular (Fo) B cells (10e5 cells/well) were cultured with LPS (5 µg/ml) or α-CD40 in the presence or absence of anti-IL-10R antibody (1 µg/ml) for 48 hours. Cell proliferation was determined by 3[H] thymidine incorporation.
(0.65 MB TIF)

Figure S6 B-2S cells (10e5 cells/well) were cultured with LPS or α-CD40 in the presence or absence of anti-IL-10R antibody (1 µg/ml) for 48 hours and cell cycle analysis was performed by PI staining.
(0.24 MB TIF)
