# Supplementary figures and images for: Interleukin-10 Mediated Autoregulation of Murine B-1 B-Cells and Its Role in Borrelia hermsii Infection
Source: PLoS One. 2010 Jul 6;5(7):e11445. doi: 10.1371/journal.pone.0011445 (PMC2897882; doi:10.1371/journal.pone.0011445)

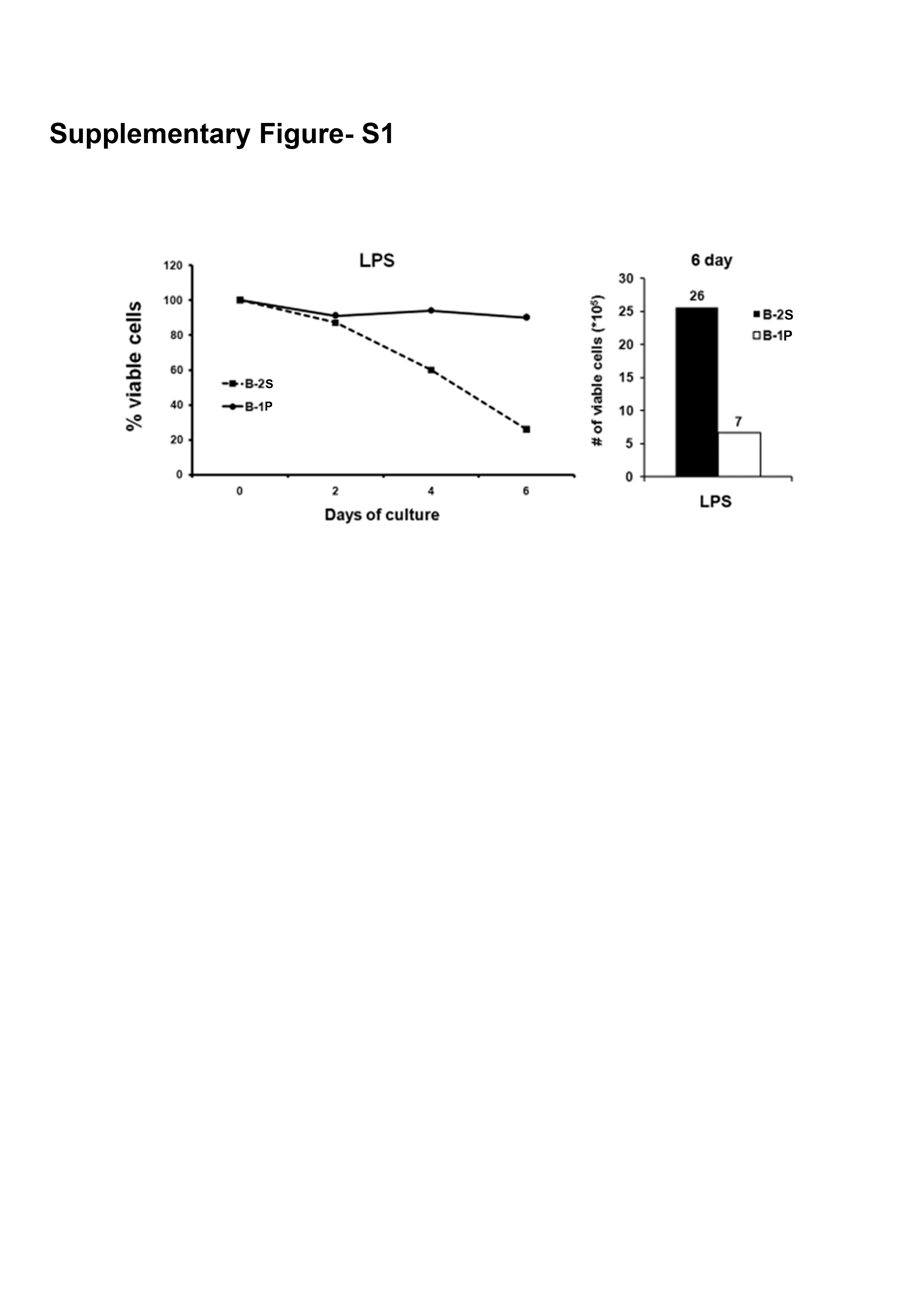

Supplement: Figure S1 — B2S and B1P were plated at a cell density of 10e5 cells/well and stimulated with LPS (5 µg/ml). Cell viability was measured at days -2, 4 and 6 by the trypan blue dye exclusion method. (0.19 MB TIF) [file pone.0011445.s001.tif]

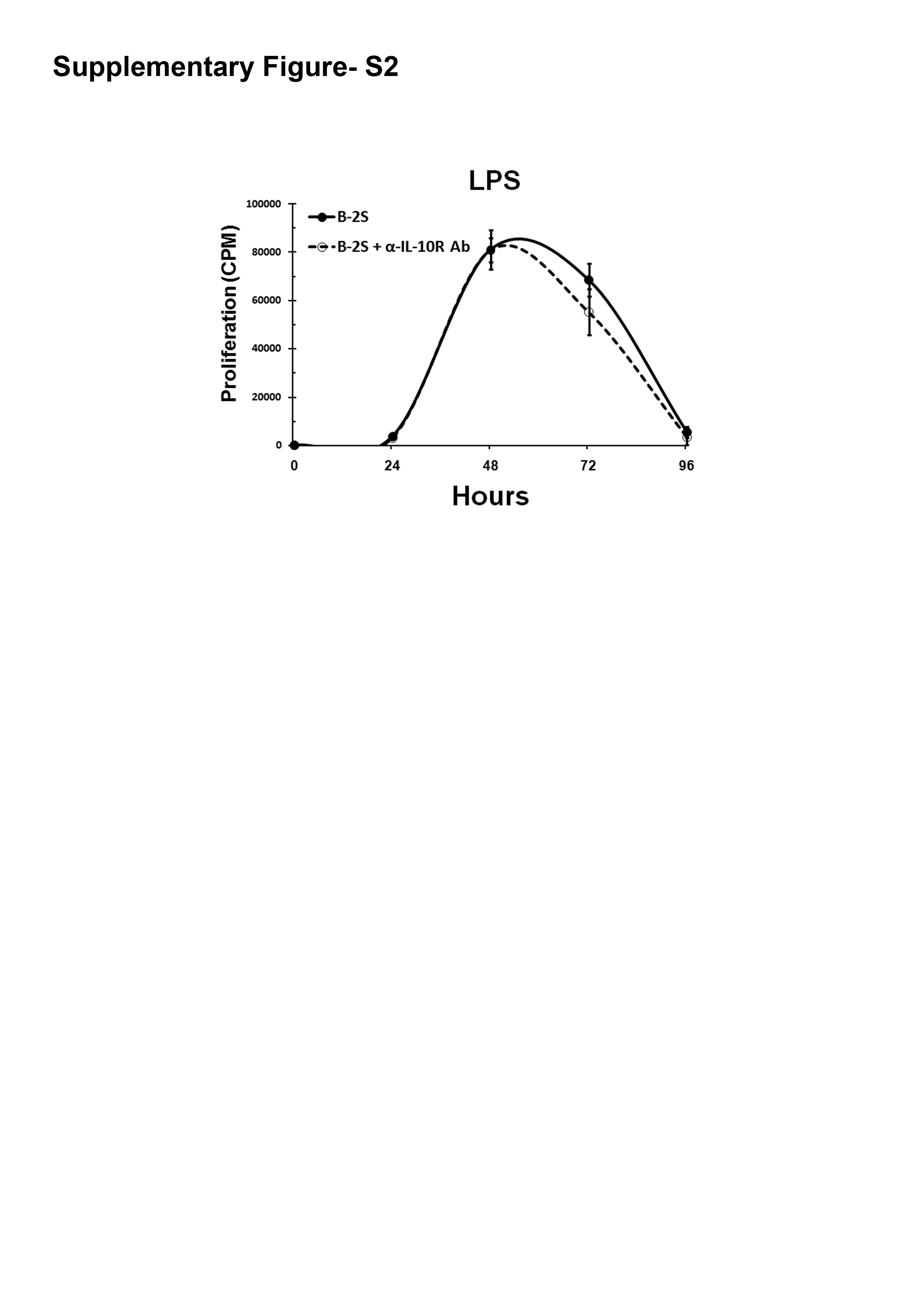

Supplement: Figure S2 — B-2S cells (10e5 cells/well) were cultured with LPS (5 µg/ml) in the presence or absence of anti-IL-10R antibody (1 µg/ml); proliferation was measured by 3[H] thymidine incorporation. (0.14 MB TIF) [file pone.0011445.s002.tif]

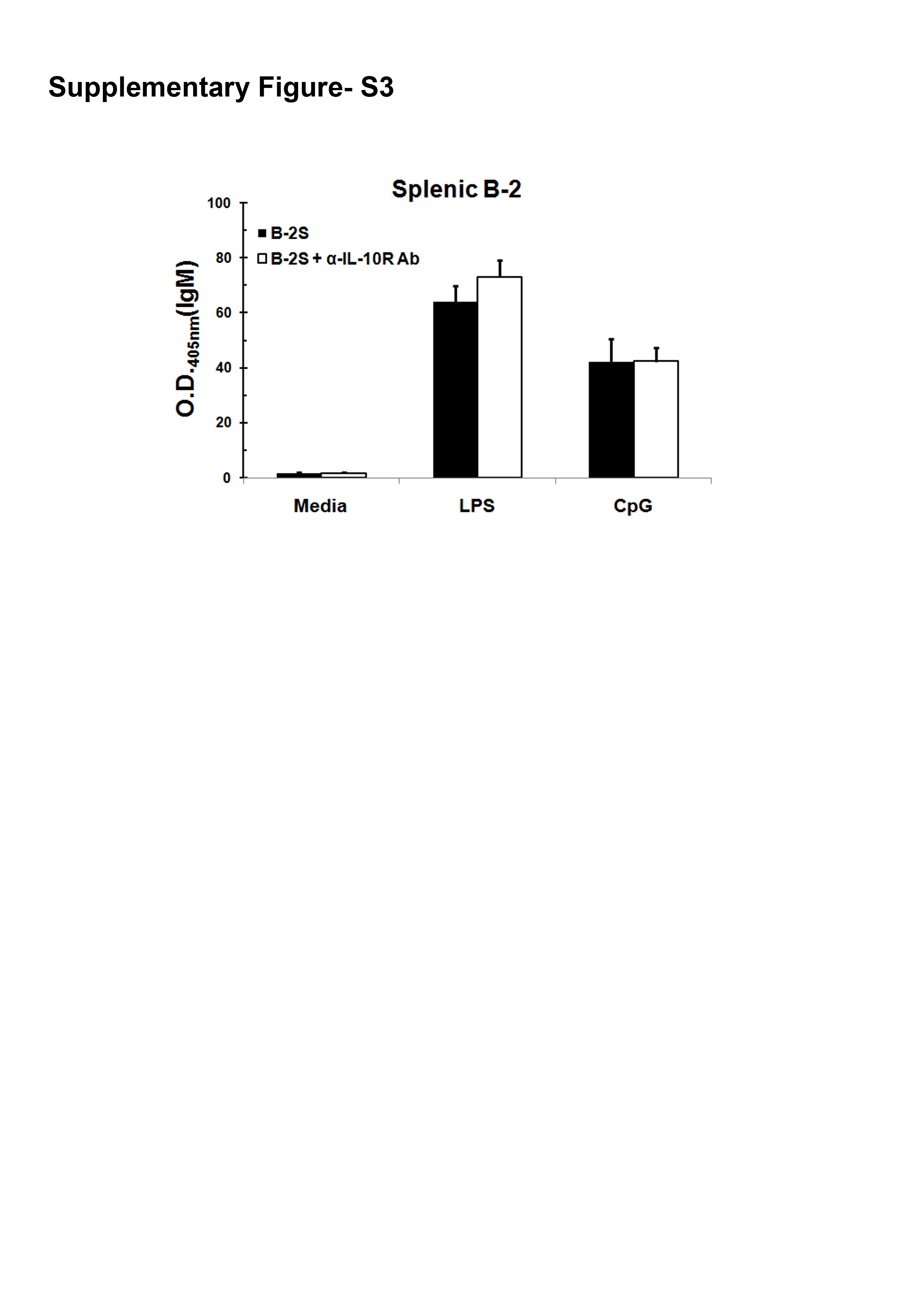

Supplement: Figure S3 — B-2S cells (10e5 cells/well) were cultured with LPS (5 µg/ml) or CpG (5 µg/ml) for 5 days in the presence or absence of anti-IL-10R antibody (1 µg/ml). At the end of 5 days culture supernatants were collected and assayed by ELISA for total IgM. (0.29 MB TIF) [file pone.0011445.s003.tif]

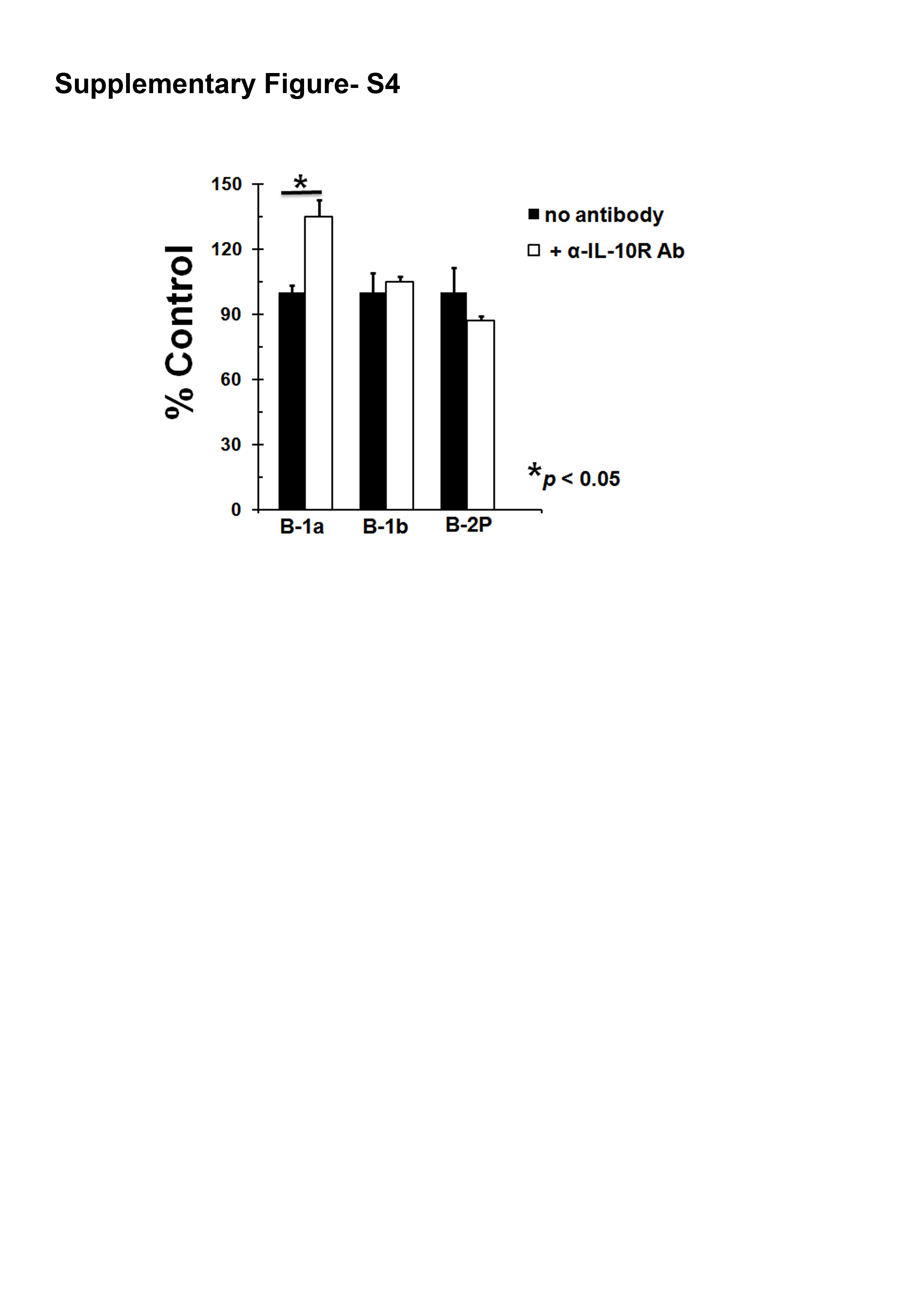

Supplement: Figure S4 — FACS sorted peritoneal B-1a, B-1b and B-2P cells (10e5 cells/well) were cultured with LPS (5 µg/ml) in the presence or absence of anti-IL-10R antibody (1 µg/ml) for 48 hours. Cell proliferation was determined by 3[H] thymidine incorporation. (0.32 MB TIF) [file pone.0011445.s004.tif]

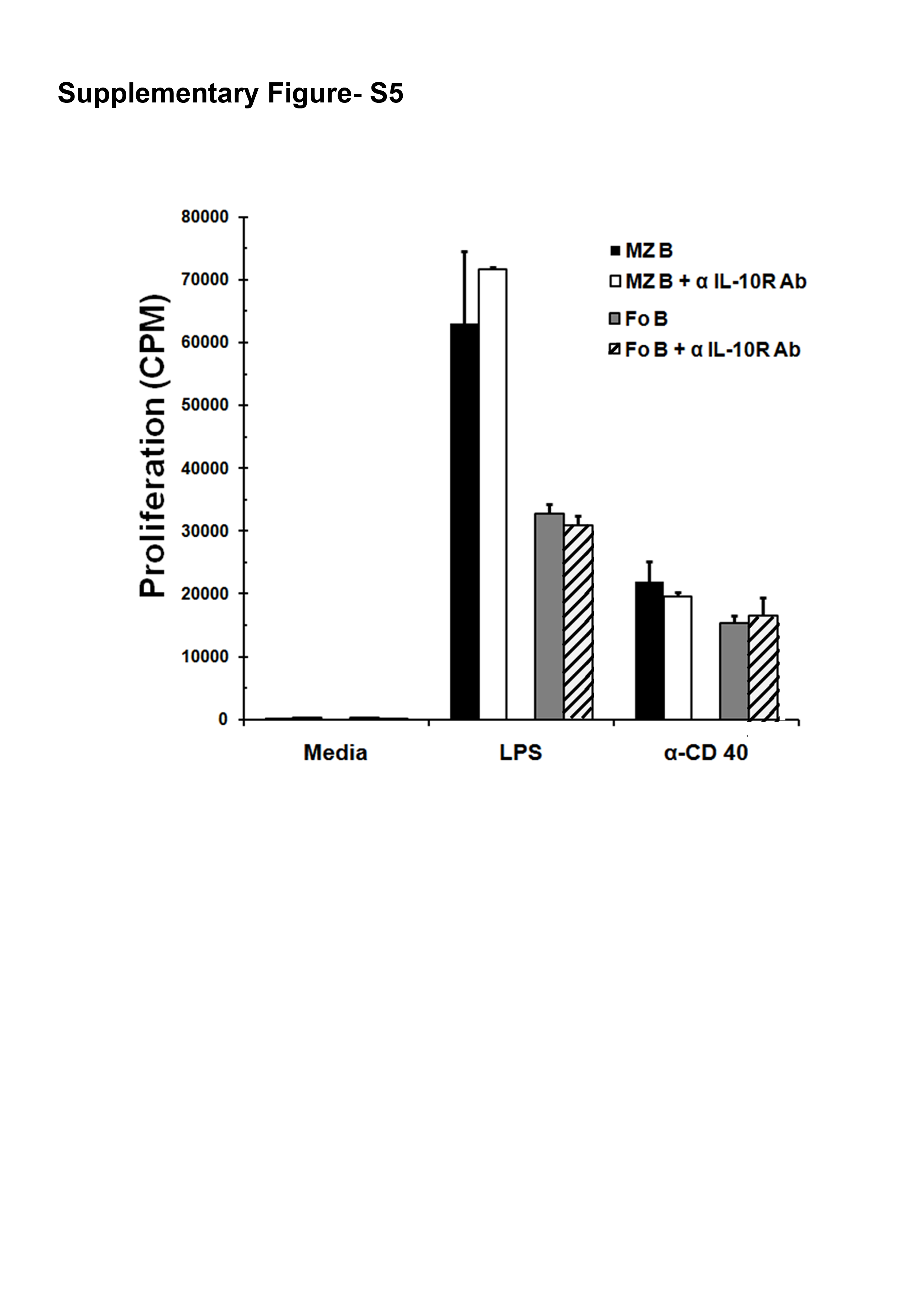

Supplement: Figure S5 — FACS sorted splenic marginal zone (MZ) and follicular (Fo) B cells (10e5 cells/well) were cultured with LPS (5 µg/ml) or α-CD40 in the presence or absence of anti-IL-10R antibody (1 µg/ml) for 48 hours. Cell proliferation was determined by 3[H] thymidine incorporation. (0.65 MB TIF) [file pone.0011445.s005.tif]

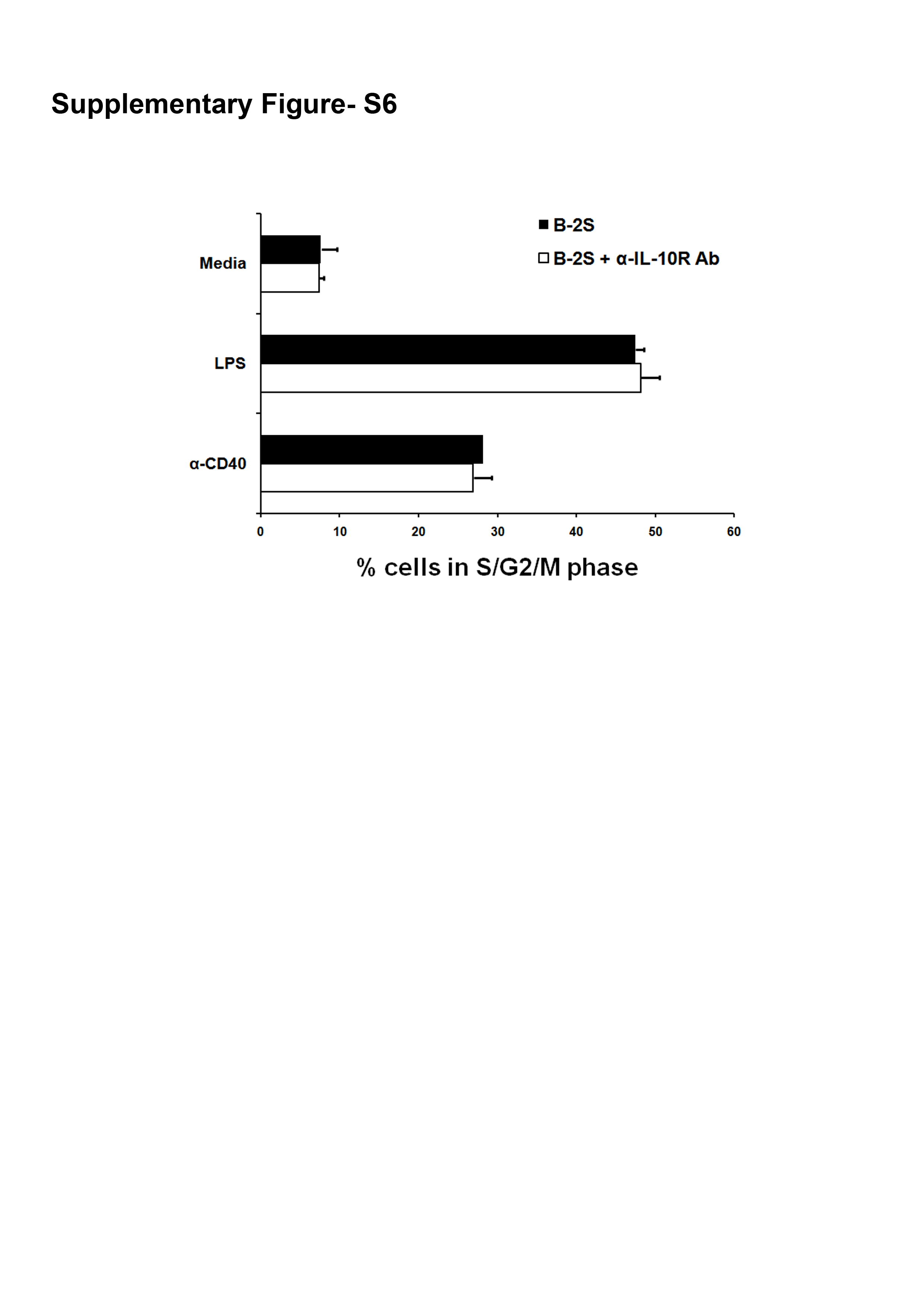

Supplement: Figure S6 — B-2S cells (10e5 cells/well) were cultured with LPS or α-CD40 in the presence or absence of anti-IL-10R antibody (1 µg/ml) for 48 hours and cell cycle analysis was performed by PI staining. (0.24 MB TIF) [file pone.0011445.s006.tif]
